# Comparison of ATG-thymoglobulin with atg-fresenius in patients with hematological malignancies who undergo allogeneic hematopoietic stem cell transplantation: a propensity score-matched analysis

**DOI:** 10.1007/s00277-025-06267-4

**Published:** 2025-02-28

**Authors:** Hanyue Zhang, Yuhang Zhou, Kui Zhao, Jiaqi Cui, Xiangzhong Zhang, Ruijuan Wen, Yanling Sun, Xudong Li, Bing Long

**Affiliations:** 1 Department of Hematology, The Third Affiliated Hospital, Sun Yat-sen University, Guangzhou, China; 2 Department of Gastroenterology, The Eighth Affiliated Hospital, Sun Yat-sen University, Shenzhen, China

**Keywords:** ATG-thymoglobulin, ATG-fresenius, Allogeneic hematopoietic stem cell transplantation, Propensity score-matched analysis, GVHD

## Abstract

We retrospectively compared the outcomes of 166 patients with hematological malignancies who underwent allogeneic hematopoietic stem cell transplantation (allo-HSCT) using ATG-Thymoglobulin (ATG-T) at 10 mg/kg or ATG-Fresenius (ATG-F) at 20 mg/kg. Propensity score matching (PSM) analysis was applied, with 44 patients assigned to each group. The ATG-T group showed a trend toward a higher incidence of bacterial infections (72.7% vs. 65.9%, *P* = 0.064). Additionally, the ATG-T group had a significantly higher incidence of other viral infections, including BK virus and herpes zoster virus (40.9% vs. 15.9%, *P* = 0.003), compared to the ATG-F group. Furthermore, the ATG-F group experienced a lower incidence of high fever (4.5% vs. 50.0%, *P* < 0.001) and reduced ATG treatment costs [¥ 45100 (28700–82000) vs. ¥ 56250 (38000–85000), *P* < 0.001] compared to ATG-T. The incidences of acute GVHD, grade III-IV aGVHD, grades of aGVHD, chronic GVHD, 3-year overall survival (OS), transplantation-related mortality (TRM), non-relapse mortality (NRM), disease-free survival (DFS), and GVHD-free and relapse-free survival (GRFS) were similar between the ATG-T and ATG-F groups. In conclusion, our study suggests that ATG-F is superior to ATG-T in terms of viral infections, fever rate, and treatment cost.

**Supplementary Information:**

The online version contains supplementary material available at 10.1007/s00277-025-06267-4.

## Introduction

Allogeneic hematopoietic stem cell transplantation (allo-HSCT) is an effective and potentially curative treatment for many hematologic malignancies. However, complications including graft-versus-host disease (GVHD) significantly increase the rate of transplantation-related morbidity and mortality. Currently, over 20 medications are used worldwide for GVHD prophylaxis [1–2]. The prevention strategy of GVHD consists of three aspects, including pharmacologic prophylaxis, serologic prophylaxis, and graft preconditioning prophylaxis. Pharmacological prophylaxis typically involves a combination of calcineurin inhibitors with methotrexate or mTOR inhibitors and mycophenolate mofetil (MMF), while post-transplant cyclophosphamide is emerging as a promising approach for GVHD prevention [3]. Despite these advances, the incidence of acute GVHD (aGVHD) remains at 30–50%, with severe aGVHD (grades 3–4) occurring in about 15% of cases. Additionally, the incidence of chronic GVHD (cGVHD) ranges from 30 to 70% [4–6].

Numerous studies have shown that anti-thymocyte globulins (ATGs) are commonly used to reduce the incidence of cGVHD in haploidentical hematopoietic stem cell transplantation (HID-HSCT) and matched unrelated donor (MUD) transplantation, leading to improved survival outcomes. However, the use of ATG may lead to increased infection and relapse rates [7–17]. ATG targets immune system cells through multiple mechanisms, including T-cells [18–19]. Two commercial preparations of rabbit ATG are widely used: ATG-Thymoglobulin (rabbit ATG-Genzyme, ATG-T), primarily for hematopoietic stem cell transplantation, and ATG-Fresenius (rabbit ATG-Fresenius, ATG-F), more commonly used in organ transplantation [20–22]. ATG-T is produced by immunizing rabbits with human thymocytes, while ATG-F is produced by immunizing rabbits with the Jurkat human T-lymphoblastoid cell line [23]. Due to differences in their manufacturing processes, these two products contain distinct antibody specificities and quantities, leading to variations in lymphocyte depletion efficacies between ATG-T and ATG-F [23].

Several studies have compared the protective effects of ATG-T and ATG-F in allo-HSCT, but the results remain inconclusive. In some studies, the dosages of the two products were not standardized, making the findings difficult to interpret [24–25]. Other studies that compared fixed doses of ATG-T and ATG-F may have been affected by differences in pretreatment regimens, ATG brands, dosages, or sample sizes, limiting the ability to draw definitive conclusions [26–28]. Therefore, further research is needed to compare the efficacy of ATG-T and ATG-F using fixed doses.

To address this issue, we retrospectively analyzed data from 166 patients who underwent haplo-HSCT and unrelated HSCT at our center, using a propensity score matched (PSM) cohort study. These patients received either 10 mg/kg ATG-T or 20 mg/kg ATG-F during transplantation. We compared the incidence of cGVHD, aGVHD, engraftment rates, CMV and EBV reactivation rates, infection rates (including bacterial, BK virus and herpes zoster virus, and fungal infections), 100-day transplantation-related mortality, overall survival, relapse rate, non-relapse mortality, disease-free survival and GVHD-free and relapse-free survival. Our objective was to optimize the GVHD prevention regimens, reduce both acute and chronic GVHD incidences, and provide a clinical basis for improving transplantation efficacy. Appropriate statistical analyses were used to compensate for imbalances in baseline characteristics.

## Materials and methods

### Patients

We conducted a retrospective study of 166 patients who underwent MUD-HSCT and HID-HSCT at the Third Affiliated Hospital of Sun Yat-sen University between August 2012 and November 2023. All procedures followed the Helsinki Declaration, and informed consent was obtained from all patients for the analysis of their clinical data. The study protocol was approved by the ethics committee of the Third Affiliated Hospital of Sun Yat-sen University. Eligibility for allo-HSCT required normal heart, lung, liver, and renal function, as determined by routine clinical and laboratory evaluations.

### Transplantation procedure

All patients received a myeloablative conditioning (MAC) regimen, either TBI/CY or Bu/Cy. For patients receiving the TBI/CY regimen, a total of 12 Gy was administered on days − 7 to -6, along with 120 mg/kg cyclophosphamide. For those receiving the Bu/Cy regimen, busulfan (3.2 mg/kg/day on days − 8 to -6), and cyclophosphamide (60 mg/kg/day on days − 3 to -2) were administered. Peripheral blood stem cells were used for patients undergoing MUD-HSCT, while patients undergoing HID HSCT received both bone marrow and peripheral blood stem cells. In cases of major ABOi-HSCT with an anti-donor isohemagglutinins (ISO) titer of ≥ 1:32, the volume of RBCs in peripheral blood grafts should be < 20 mL, and RBCs in bone marrow grafts should be depleted. In cases of minor ABOi-HSCT with an anti-recipient ISO titer of ≥ 1:256, plasma depletion of both peripheral blood grafts and bone marrow grafts is necessary [29]. All patients were given subcutaneous rhG-CSF (5 mg/kg/day) starting on day 3 post-transplantation and continued until myeloid recovery.

For GVHD prophylaxis, patients received either ATG-T (2.5 mg/kg/day on days − 4 to -1) or ATG-F (5 mg/kg/day on days − 4 to -1). In addition, patients were administered cyclosporine (CSA, 2.5 mg/kg/day starting on day − 1, with serum levels maintained at 250–300 ng/mL), methotrexate (15 mg/m² on day + 1 and 10 mg/m² on days + 3 and + 6), and mycophenolate mofetil (MMF, 500 mg/day from day − 1 to + 28).

For infection prevention, oral acyclovir was given to prevent herpesvirus, posaconazole or intravenous caspofungin were used for fungal infection prophylaxis, and oral cotrimoxazole was administered to prevent Pneumocystis carinii. Routine prophylaxis for hepatic veno-occlusive disease (VOD) included prostaglandin and reduced glutathione.

CMV-DNA and EBV-DNA levels were monitored weekly post-transplant using polymerase chain reaction (PCR). If CMV-DNA levels exceeded 1000 copies/mL, preemptive treatment with ganciclovir or foscarnet sodium, combined with immunoglobulin, was initiated. If ganciclovir and foscarnet proved ineffective, cidofovir was administered. Rituximab was used to treat post-transplant lymphoproliferative disease in patients with elevated EBV-DNA levels.

### Study design and efficacy measurements

The primary endpoint was the incidence of acute and chronic GVHD, while secondary endpoints included engraftment rates, infection rates (bacterial, fungal, CMV, EBV and other virus), 100-day transplant-related mortality (TRM), relapse rates, overall survival (OS), non-relapse mortality (NRM), disease-free survival (DFS), and GVHD-free relapse-free survival (GRFS).

The diagnosis and grading of aGVHD and cGVHD were based on established criteria. According to the 1994 Consensus Conference on Acute GVHD Grading, aGVHD was graded from 0 to 4, while cGVHD was classified as mild, moderate, or severe following the NIH Working Group criteria [30–31].

Neutrophil engraftment was defined as the first of three consecutive days with an absolute neutrophil count of ≥ 0.5 × 10^9^/L. Platelet engraftment was defined as the first of five consecutive days with a platelet count of ≥ 20 × 10^9^/L without transfusion support.

We defined OS and disease relapse as death from any cause, and any evidence of recurrence if they were in remission at time of transplant, respectively. And TRM was defined as death resulting from any complication directly attributable to the transplantation process, excluding disease relapse. NRM refers to mortality due to non-relapse causes, and DFS was defined as the time from transplantation to disease relapse or recurrence. We defined GRFS as the time from transplantation during which patients remained free from grade III-IV acute GVHD (aGVHD), chronic GVHD (cGVHD) requiring systemic immunosuppressive therapy.

CMV and EBV reactivation were defined as DNA levels > 500 copies/mL at two consecutive time points.

### Statistical analysis

Categorical variables were analyzed using the chi-square test or Fisher’s exact test, while continuous variables were compared with the independent sample t-test. And the cumulative incidence functions were used to estimate aGVHD, cGVHD, engraftment rates, infection rates, relapse rates and NRM in a competing risk setting. Given the study design, baseline characteristics imbalances were expected, and the following statistical methods were applied to account for them: (1) univariate and multivariate analyses adjusted for potential confounders, (2) propensity score matching (PSM). A 1:1 propensity score match with a caliper value of 0.05 was performed using matching criteria such as donor relatedness, age, gender, diagnosis, disease risk index, pre-transplant status, HCT-CI score, CMV serology status of recipients, donor-recipient gender match, the year of transplant and use of letermovir. A *P* value < 0.05 was considered statistically significant and (3) in our study, multiple outcome events (such as NRM, death, and relapse) may occur simultaneously, and these events are mutually competitive. Traditional survival analysis methods, such as Cox regression, do not adequately address these competing risks. The use of a competing risks model offers a more accurate estimation of the incidence of each event and its associated risk factors, providing a more precise risk assessment. SPSS version 25.0 and R version 4.4.2 for Windows were used for statistical analysis, and Kaplan–Meier plots were generated using GraphPad Prism.

We compared the distribution of the following variables before and after propensity score matching (PSM) using propensity score distribution plots: donor relatedness, age, gender, diagnosis, disease risk index, pre-transplant status, HCT-CI score, CMV serology status of recipients, donor-recipient gender match, year of transplant, and use of letermovir. This approach allows us to assess the balance of these key clinical characteristics between groups before and after matching, demonstrating the adequacy of the matching and ensuring comparability for subsequent analyses.

## Results

### Patient characteristics

A total of 166 patients with hematologic malignancies were included in this retrospective study (Supplementary Table 1). The baseline characteristics of patients after PSM are listed in Table 1. Overall, the two groups were well-matched in terms of demographics and physical characteristics, including age, gender, diagnosis, disease risk index, pre-transplant status, HCT-CI score, stem cell source, CMV serology status of donors and recipients, donor relatedness, donor-recipient gender match, donor-recipient ABO blood group match, time from diagnosis to transplantation, the year of transplant, use of letermovir and conditioning regimen.

**Table 1:** Basic characteristics of patients

	HID + Unrelated (*n* = 88)		
	ATG-T (10 mg/kg, *n* = 44)	ATG-F (20 mg/kg, *n* = 44)	*P* Value
Age, years, median (range)	28 (12–53)	29.5 (13–59)	0.878
Gender, male (%)	27 (61.4)	27 (61.4)	1
Diagnosis, N (%)			0.160
AML ALL MDS Others	20 (45.5) 18 (40.9) 6 (13.6) 0 (0)	26 (59.1) 12 (27.3) 4 (9.1) 2 (4.5)	
Disease risk index, N (%)			0.412
Low risk Intermediate risk High risk Very high risk	4 (9.1) 12 (27.3) 27 (61.4) 1 (2.3)	1 (2.3) 17 (38.6) 25 (56.8) 1 (2.3)	
Pre-transplant status, N (%)			1
CR NR	36 (81.8) 8 (18.2)	36 (81.8) 8 (18.2)	
HCT-CI			0.210
0 1 2	30 (68.2) 12 (27.3) 2 (4.5)	29 (65.9) 15 (34.1) 0 (0)	
Stem cell source, N (%)			0.286
BM + PB PB	25 (56.8) 19 (43.2)	24 (54.5) 20 (45.5)	
CMV serology status of donors			0.296
Positive Negative	43 (97.7) 1 (2.3)	41 (93.2) 3 (6.8)	
CMV serology status of recipients			1
Positive Negative	44 (100.0) 0 (0)	44 (100.0) 0 (0)	
Donor relatedness, N (%)			0.177
HID Unrelated	26 (59.1) 18 (40.9)	32 (72.7) 12 (27.3)	
Donor-recipient gender match, N (%)			1
Match Mismatch	26 (59.1) 18 (40.9)	26 (59.1) 18 (40.9)	
Donor-recipient ABO blood group match, N (%)			0.521
Match Mismatch	19 (43.2) 25 (56.8)	22 (50.0) 22 (50.0)	
Time from diagnose to transplantation, median (range)	183 (92–753)	185 (81-4595)	0.073
The year of transplant, median (range)	2017 (2012–2023)	2018 (2015–2022)	1
Use of letermovir, N (%)	1 (2.3)	0 (0)	1
Conditioning regimen, N (%)			1
BU + CY TBI + CY	41 (93.2) 3 (6.8)	41 (93.2) 3 (6.8)	

AML, acute myeloid leukemia; ALL, acute lymphoblastic leukemia; MDS, myelodysplastic syndromes; CR, complete remission; NR, no response; HCT-CI, Hematopoietic Cell Transplantation-Comorbidity Index; PB, peripheral blood; BM, bone marrow; HID, Haploidentical donor; BU, busulfan; TBI, total body irradiation; CY, cyclophosphamide

### Engraftment

Neutrophil engraftment was observed in all patients in the ATG-T group, while one patient in the ATG-F group experienced neutrophil engraftment failure (*P* = 0.333, Supplementary Table 2). There were no significant differences in neutrophil engraftment time (11.70 days vs. 11.91 days, *P* = 0.654, Supplementary Table 2) or platelet engraftment time (14.47 days vs. 16.09 days, *P* = 0.329, Supplementary Table 2) between the ATG-T and the ATG-F groups. Cumulative incidence of platelet engraftment by day 100 was identical between the two groups (97.7% vs. 97.7%, *P* = 0.579, Supplementary Table 2).

### GVHD

**Fig. 1:**
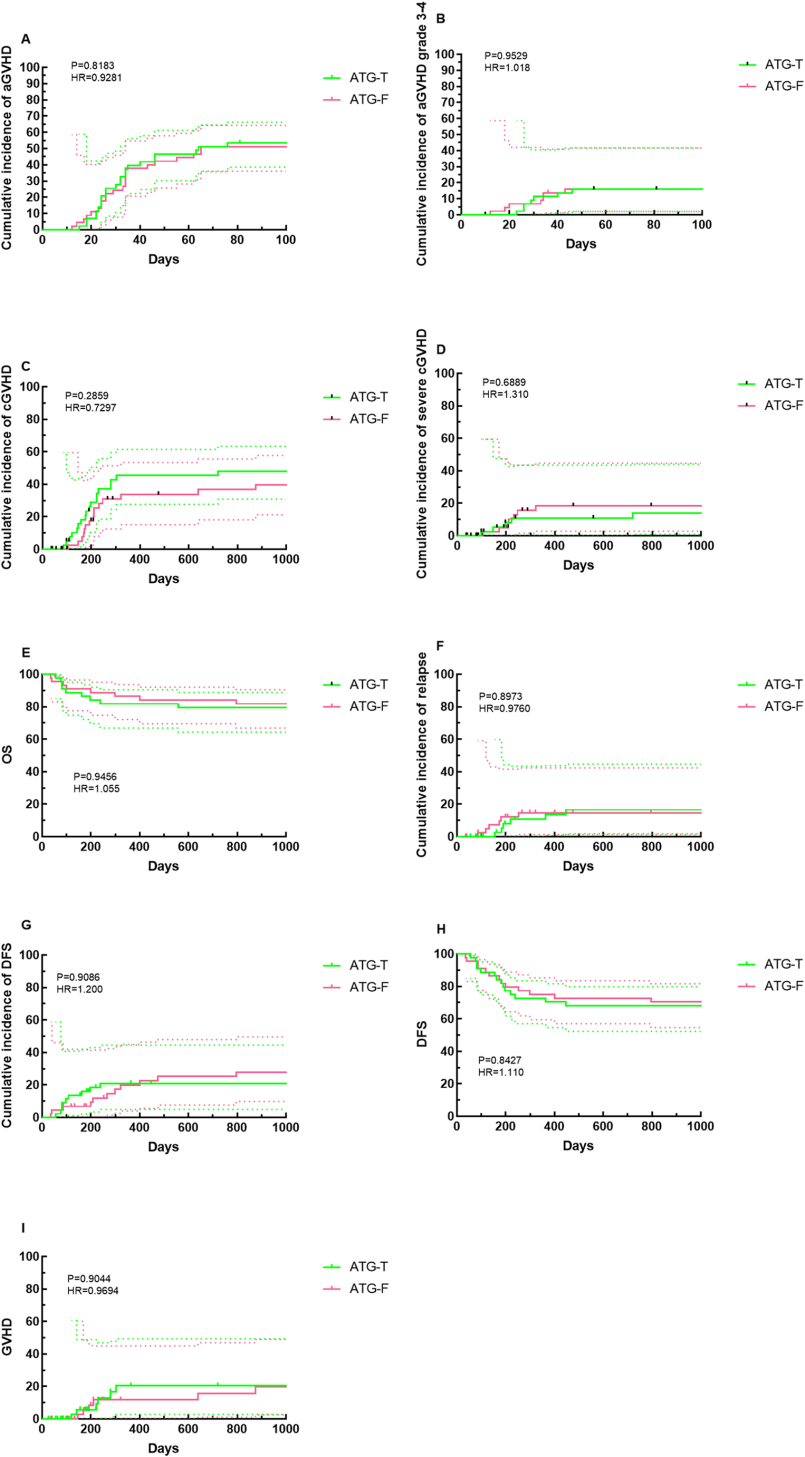
Clinical outcomes of the two groups. **A** Cumulative incidence of aGVHD based on ATG type. **B** Cumulative incidence of aGVHD grade 3–4 based on ATG type. **C** Cumulative incidence of cGVHD based on ATG type. **D** Cumulative incidence of severe cGVHD based on ATG type. **E** Outcomes of survival based on ATG type. **F** Cumulative incidence of relapse based on ATG type. **G** Cumulative incidence of non-relapse mortality (NRM) based on ATG type. **H** Disease-free survival (DFS) based on ATG type. **I** GVHD-free relapse-free survival (GRFS) based on ATG type

There was no significant difference in the cumulative incidence of aGVHD between the ATG-T and ATG-F groups (54.5% vs. 50.0%, *P* = 0.643, Supplementary Table 3), nor in the incidence of grade 3–4 aGVHD (15.9% vs. 15.9%, *P* = 0.983, Supplementary Table 3). The cumulative incidence curves of aGVHD and grade 3–4 aGVHD were close to overlapping at 100 days (Fig. 1). Univariate analysis showed that the type of ATG preparation had no significant impact on the cumulative incidence of grade 3–4 aGVHD (*P* = 0.103, Supplementary Table 5).

Regarding cGVHD, 18 patients in the ATG-T group (40.9%) developed cGVHD, with 5 cases classified as severe (11.4%). In the ATG-F group, 15 patients (34.1%) developed cGVHD, including 7 severe cases (15.9%). The median time to cGVHD onset was 205 days (156–447) in the ATG-T group and 153 days (86–253) in the ATG-F group (*P* = 0.085, Supplementary Table 3). At 3 years post-transplant, the cumulative incidence of total cGVHD was not significantly different between the ATG-T and ATG-F groups (40.9% vs. 34.1%, *P* = 0.447, Supplementary Table 3). Similarly, there was no significant difference in the cumulative incidence of severe cGVHD at 3 years (11.4% vs. 15.9%, *P* = 0.261, Supplementary Table 3). The cumulative incidence curves of cGVHD and severe cGVHD showed different trends. The cumulative incidence of cGVHD was higher in the ATG-T group than in the ATG-F group after 1 year post-transplantation. However, for severe cGVHD, the cumulative incidence was higher in the ATG-F group than in the ATG-T group. However, these differences did not reach statistical significance (Fig. 1). In univariate analysis, factors such as age ≥ 40 years was associated with cGVHD occurrence. However, multivariate analysis showed that age ≥ 40 years was not a risk factor for the development of cGVHD.

### Relapse and survival

There was no statistically significant difference in 3-year OS between the ATG-T and ATG-F groups (72.7% vs. 70.5%, *P* = 0.813, Supplementary Table 4). 6 patients in the ATG-T group and 6 in the ATG-F group experienced post-transplant relapse. The 3-year cumulative incidence of relapse (CIR) was not significantly different between the ATG-T and ATG-F groups (13.6% vs. 13.6%, *P* = 0.933, Supplementary Table 4). The median time to recurrence was also similar between the two groups (205 days vs. 153 days post-transplant, *P* = 0.085, Supplementary Table 3). TRM did not differ significantly between the ATG-T and ATG-F groups (11.4% vs. 9.1%, *P* = 0.725, Supplementary Table 4). The 3-year cumulative incidence of non-relapse mortality (NRM) was not significantly different between the ATG-T and ATG-F groups (20.5% vs. 25.0%, *P* = 0.720, Supplementary Table 4). Likewise, there were no significant differences in 3-year DFS (65.9% vs. 61.4%, *P* = 0.658, Supplementary Table 4) or 3-year GRFS (50.0% vs. 52.3%, *P* = 0.831, Supplementary Table 4). The trends in the cumulative incidence curves for OS, relapse, DFS, NRM, and GRFS were close to identical in ATG-Tand ATG-F groups (Fig. 1).

### Infections and adverse events

The ATG-T group showed a trend toward a higher cumulative incidence of bacterial infections (72.7% vs. 65.9%, *P* = 0.064, Table 2). Additionally, the ATG-T group had a significantly higher cumulative incidence of other viral infections, including BK virus and herpes zoster virus (40.9% vs. 15.9%, *P* = 0.003, Table 2), compared to the ATG-F group. The cumulative incidence of CMV reactivation (55.9% vs. 56.8%, *P* = 0.193, Table 2), EBV reactivation (4.5% vs. 0%, *P* = 0.155, Table 2) and fungal infections (50.0% vs. 40.9%, *P* = 0.173, Table 2) were similar between the two groups. Furthermore, the ATG-F group had a significantly lower incidence of high fevers (≥ 39℃) compared to the ATG-T group (4.5% vs. 50.0%, *P* < 0.001, Table 2). Also, the duration of fever was significantly longer in the ATG-T group than in the ATG-F group (3.79 days vs. 2.19 days, *P* = 0.011, Table 2). However, there was no significant differences in the rate of septic shock between the two groups.

**Table 2:** Infectious complications after HSCT

	HID + Unrelated (*n* = 88)		
	ATG-T (10 mg/kg, *n* = 44)	ATG-F (20 mg/kg, *n* = 44)	*P* Value
T ≥ 39℃, N (%)	22 (50.0)	2 (4.5)	***<0.001***
Duration of fever, days, average (range)	3.79 (1–12)	2.19 (1–10)	***0.011***
Septic shock, N (%)	4 (9.1)	2 (4.5)	0.393
Cumulative incidence of cytomegalovirus reactivation by 3 years (%)	55.9	56.8	0.193
Cumulative incidence of Epstein–Barr virus reactivation by 3 years (%)	4.5	0	0.155
Cumulative incidence of bacterial infection by 3 years (%)	72.7	65.9	0.064
Cumulative incidence of fungus infection by 3 years (%)	50.0	40.9	0.173
Cumulative incidence of other virus infection by 3 years (%)	40.9	15.9	***0.003***

Other virus infection, BK virus and herpes zoster virus infection

### Cost of ATG

Medical insurance in China does not cover the cost of ATG. The cost of ATG treatment was significantly lower for patients receiving ATG-F compared to those receiving ATG-T [¥ 45,100 (28,700 − 82,000) vs. ¥ 56,250 (38,000–85,000), *P* < 0.001, Table 3].

**Table 3:** Cost on ATG after HSCT

	HID + Unrelated (*n* = 88)		
	**ATG-T (10 mg/kg**,*n*** = 44)**	**ATG-F (20 mg/kg**,*n*** = 44)**	*P* Value
Cost on ATG, ¥, median (range)	56,250 (38000–85000)	45,100 (28700–82000)	***<0.001***

## Discussion

GVHD is a major complication of allo-HSCT and has become one of the leading causes of post-transplant mortality, significantly impacting patients’ quality of life [10, 32]. Despite advancements in GVHD prophylaxis therapies over the past few decades, the improvement in outcomes remains limited [33–35]. The traditional standard prophylaxis for aGVHD typically consists of a calcineurin inhibitor, such as cyclosporine (CSA) or tacrolimus (TAC), combined with an antimetabolite, such as mycophenolate mofetil (MMF) or methotrexate (MTX) [36]. The persistence of severe aGVHD has stimulated numerous studies aimed at improving prevention strategies. One of the most extensively studied approaches is T-cell depletion (TCD), which involves selectively removing donor T cells from the graft using monoclonal antibodies (e.g., alemtuzumab) or polyclonal antisera (e.g., antithymocyte globulin [ATG]) [37]. An alternative to selective T-cell depletion is post-transplant cyclophosphamide (PTCy). Massoud and colleagues developed a proprietary formulation of ATG (ATLG) and compared it to post-transplant PTCy in patients undergoing allogeneic myeloablative HSCT. The authors found that while both strategies effectively reduced GVHD, ATLG’s stronger immunosuppressive effects might delay immune recovery, potentially increasing the risk of infections [38].

For cGVHD, two main strategies are used for prevention: selecting the type of allogeneic graft (bone marrow vs. peripheral blood stem cells) and T-cell depletion. Studies suggest that bone marrow transplantation is associated with a lower risk of cGVHD compared to peripheral blood stem cell transplantation [39–41]. Additionally, T-cell depletion has been shown to reduce the risk of cGVHD, with the strongest evidence coming from studies involving ATG [10, 42]. Research indicates that ATG can reduce the incidence of both aGVHD and cGVHD, making it a promising component of prophylactic regimens, especially in high-risk or mismatched transplants. However, the optimal dosing and formulation of ATG remain active areas of investigation.

The two types of rabbit ATG currently used, ATG-T and ATG-F differ in pharmacokinetic and pharmacodynamic properties due to distinct manufacturing processes. These polyclonal IgGs are derived from rabbit immune sera, with ATG-T targeting human thymocytes and ATG-F targeting the Jurkat T-cell leukemia line [43–46]. ATG-T binds a broader range of antigens, including T cells (CD2, CD3, CD4, CD6, CD8), B cells, natural killer cells, macrophages, dendritic cells, HLA-I, and HLA-DR. In contrast, ATG-F binds fewer antigens and lacks antibodies against CD3, CD4, or HLA-DR [47].

Although numerous studies support the efficacy of ATG in preventing GVHD, findings regarding the effectiveness of different ATG formulations have been inconsistent [24, 28]. A meta-analysis conducted by Gagelmann et al. compared different rabbit anti-thymocyte globulin formulations and concluded that the effects of different ATG are not directly equivalent. The study emphasized that selecting the appropriate ATG formulation and dosage should be tailored to the specific needs of each patient [48]. In this retrospective study, we analyzed the outcomes of ATG-T (10 mg/kg) versus ATG-F (20 kg/mg) in haplo-HSCT and unrelated HSCT, focusing on graft source, conditioning regimen, GVHD prophylaxis, and supportive care. No significant differences were observed in post-transplant hematopoietic recovery, acute and chronic GVHD incidence, relapse rate, OS, TRM, NRM, DFS, or GRFS after PSM.

The usage of ATG fails to reach a consensus around the globe, with different centers relying on their own clinical experience. However, a general distinction has been made between high and low dosage regimens based on different medical situation, and growing numbers of studies have paid attention to the clinical outcomes and other events associated between the different dosages. Paiano et al. conducted a randomized study comparing ATG-T (7.5 mg/kg) with ATG-F (25 mg/kg) in 30 patients undergoing Bu/Flu conditioning. They found no differences in 3-year OS, DFS, relapse, or TRM [49]. The same conclusion was achieved by another retrospective study in CIR, NRM, grade 2–4 aGVHD, cGVHD, or GRFS between the above doses [50], which was consistent with our observation. Similarly, a retrospective study by Zhou et al. compared 7.5 mg/kg of ATG-T with 20 mg/kg of ATG-F, reporting no difference in all grades of aGVHD but a higher incidence of any grade and limited cGVHD in the ATG-T group [51], but Huang et al. noted that patients receiving ATG-F (20 mg/kg) had a lower risk of developing cGVHD than those treated with ATG-T (10 mg/kg) [27]. We speculate that this is due to the fact that dose differences of ATG-T may affect the strength and persistence of the drug’s effect on T-cells and thus the incidence of cGVHD. Furthermore, Massoud et al. compared two different doses of ATLG (30 mg/kg vs. 60 mg/kg) in unrelated donor HSCTs and found that while higher doses of ATLG offer better GVHD prevention, they also come with an increased risk of infections and transplant-related mortality [52]. Higher doses of ATG have been associated with increased infection rates, TRM, and reduced OS [53–57], making the 10 mg/kg ATG-T and 20 mg/kg ATG-F doses used in our study relatively appropriate. Our results also support the reasonable application of these doses in both haplo-HSCT and unrelated HSCT.

Infections, particularly CMV and EBV reactivation, were key points of interest in our comparative study. ATG has long been recognized as a risk factor for CMV and EBV infections [58]. However, few studies directly compare infection risk between different ATG formulations. In our study, CMV and EBV reactivation rates were higher in the ATG-T group, but the difference was not statistically significant. Our result revealed that the use of ATG-T was associated with higher incidence of other viral infection, and there was a trend that ATG-T group was related to higher incidence of bacterial infection. Besides, the ATG-F group had a significantly lower incidence of high fevers (≥ 39℃) and shorter duration of fever compared to the ATG-T group, suggesting that ATG-F (20 mg/kg) may be superior to ATG-T (10 mg/kg) in terms of reducing post-transplant infection risks.

On the other hand, cost analysis showed a notable financial advantage for ATG-F. For instance, a 60 kg patient would spend nearly 2.5 times more on ATG-T than on ATG-F (¥60,000 vs. ¥24,600). Given that HSCT is a highly complex and resource-intensive procedure, with significant financial implications, particularly in the context of developing countries, medical expenditures continue to be a critical concern for both healthcare systems and patients. Our data suggest that the use of ATG-F may lead to a reduction in the costs associated with ATG therapy, thereby yielding potential economic benefits. However, the available evidence from other medical centers remains limited, underscoring the need for further research and more comprehensive evaluation.

As a retrospective analysis of 166 patients from a single center, our study still has limitations. Additionally, the heterogeneity of the patient population may restrict the generalizability of our findings. Variations in patient characteristics, such as underlying diseases, conditioning regimens, and GVHD prophylaxis strategies, could introduce biases that affect the interpretation of the outcomes. Future studies should aim to include larger, more homogeneous cohorts and adopt prospective designs to validate these findings and better assess the impact of different ATG formulations in a broader, more diverse population.

In conclusion, ATG-T (10 mg/kg) and ATG-F (20 mg/kg) appear comparable for preventing GVHD in HID-HSCT and MUD-HSCT. Both dosing regimens effectively balance outcomes, including GVHD, OS, relapse rate, TRM, DFS, GRFS, and infection rates. ATG-F, however, shows advantages in reducing bacterial infections, other viral infections, and overall cost.

## Electronic supplementary material

Below is the link to the electronic supplementary material.

Supplementary Material 1

Supplementary Material 2

## Data Availability

No datasets were generated or analysed during the current study.
