# Supplementary material for: Comparison of ATG-thymoglobulin with atg-fresenius in patients with hematological malignancies who undergo allogeneic hematopoietic stem cell transplantation: a propensity score-matched analysis
Source: Ann Hematol. 2025 Feb 28;104(3):1907–16. doi: 10.1007/s00277-025-06267-4 (PMC12031750; doi:10.1007/s00277-025-06267-4)
Supplement: Supplementary file 1 — Supplementary Material 1 [file 277_2025_6267_MOESM1_ESM.docx]

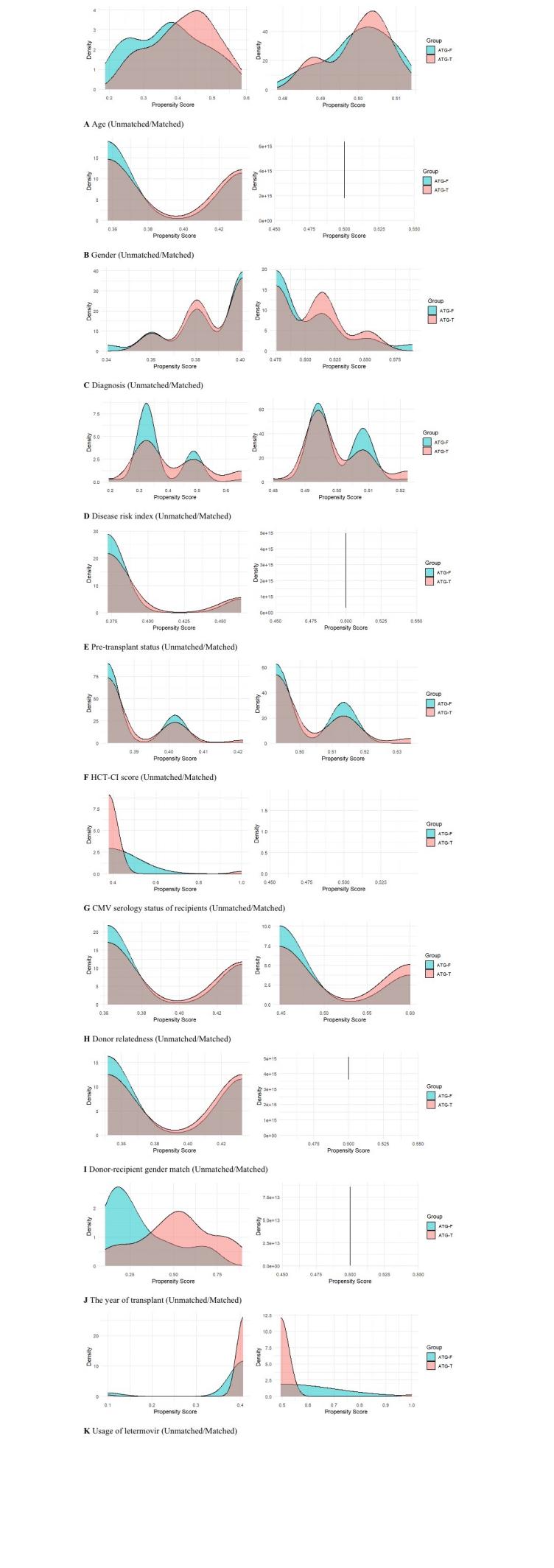


**Supplementary Figure 1:** Propensity score distribution at baseline for two types of ATG groups before and after PSM. **A** Propensity score distribution at age for two types of ATG groups before and after PSM. **B** Propensity score distribution at gender for two types of ATG groups before and after PSM. **C** Propensity score distribution at gender for two types of ATG groups before and after PSM. **D** Propensity score distribution at disease risk index for two types of ATG groups before and after PSM. **E** Propensity score distribution at pre-transplant status for two types of ATG groups before and after PSM. **F** Propensity score distribution at HCT-CI score for two types of ATG groups before and after PSM. **G** Propensity score distribution at CMV serology status of recipients for two types of ATG groups before and after PSM. **H** Propensity score distribution at donor relatedness for two types of ATG groups before and after PSM. **I** Propensity score distribution at donor-recipient gender match for two types of ATG groups before and after PSM. **J** Propensity score distribution at the year of transplant for two types of ATG groups before and after PSM. **K** Propensity score distribution at usage of letermovir for two types of ATG groups before and after PSM.
