# Supplementary material for: Comparison of ATG-thymoglobulin with atg-fresenius in patients with hematological malignancies who undergo allogeneic hematopoietic stem cell transplantation: a propensity score-matched analysis
Source: Ann Hematol. 2025 Feb 28;104(3):1907–16. doi: 10.1007/s00277-025-06267-4 (PMC12031750; doi:10.1007/s00277-025-06267-4)
Supplement: Supplementary file 2 — Supplementary Material 2 [file 277_2025_6267_MOESM2_ESM.docx]

**Supplementary Table 1:** Basic characteristics of patients before PSM

|  | **HID + Unrelated (n=166)** | | |
| --- | --- | --- | --- |
|  | **ATG-T (10mg/kg, n=64)** | **ATG-F (20mg/kg, n=102)** | **P Value** |
| Age, years, median (range) | 28 (12-53) | 35 (13-60) | ***0.006*** |
| Gender, male (%) | 35 (54.7) | 63 (61.8) | 0.367 |
| Diagnosis, N (%) AML ALL MDS Others | 33 (51.6) 23 (35.9) 8 (12.5) 0 (0) | 55 (53.9) 29 (28.4) 14 (13.7) 4 (3.9) | 0.197 |
| Disease risk index, N (%) Low risk Intermediate risk High risk Very high risk | 9 (14.1) 19 (29.7) 35 (54.7) 1 (1.6) | 2 (2.0) 27 (26.5) 69 (67.6) 4 (3.9) | ***0.014*** |
| Pre-transplant status, N (%) CR NR | 51 (79.7) 13 (20.3) | 86 (84.3) 16 (15.7) | 0.445 |
| HCT-CI 0 1 2 | 47 (73.4) 15 (23.4) 2 (3.1) | 75 (73.5) 26 (25.5) 1 (1.0) | .596 |
| Stem cell source, N (%) BM + PB PB | 25 (39.1) 39 (60.9) | 57 (55.9) 45 (44.1) | ***0.035*** |
| CMV serology status of donors  Positive  Negative | 59 (92.2) 5 (7.8) | 94 (92.2) 8 (7.8) | 0.994 |
| CMV serology status of recipients  Positive  Negative | 62 (96.9) 2 (3.1) | 102 (100.0) 0 (0) | 0.073 |
| Donor relatedness, N (%) HID Unrelated | 38 (59.4)  26 (40.6) | 68 (66.7)  34 (33.3) | 0.341 |
| Donor-recipient gender match, N (%) Match Mismatch | 32 (50.0) 32 (50.0) | 59 (57.8) 43 (42.2) | 0.323 |
| Donor-recipient ABO blood group match, N (%) Match Mismatch | 29 (45.3) 35 (54.7) | 47 (46.1) 55 (53.9) | 0.923 |
| Time from diagnose to transplantation, median (range) | 194.5 (92-10955) | 184 (15-4595) | 0.733 |
| The year of transplant, median (range) | 2017 (2012-2023) | 2015.5 (2015-2023) | ***＜0.001*** |
| Use of letermovir, N (%) | 1 (1.6) | 9 (8.8) | ***0.036*** |
| Conditioning regimen, N (%) BU + CY TBI + CY | 61 (95.3) 3 (4.7) | 94 (92.2) 8 (7.8) | 0.416 |

AML, acute myeloid leukemia; ALL, acute lymphoblastic leukemia; MDS, myelodysplastic syndromes; CR, complete remission; NR, no response; HCT-CI, Hematopoietic Cell Transplantation-Comorbidity Index; PB, peripheral blood; BM, bone marrow; HID, Haploidentical donor; BU, busulfan; TBI, total body irradiation; CY, cyclophosphamide.

**Supplementary Table 2:** Hematological recovery after HSCT

|  | **HID + Unrelated (n=88)** | | |
| --- | --- | --- | --- |
|  | **ATG-T (10mg/kg, n=44)** | **ATG-F (20mg/kg, n=44)** | **P Value** |
| Cumulative incidence of neutrophil engraftment by day 100 (%) | 100.0 | 97.7 | 0.333 |
| Cumulative incidence of platelet engraftment by day 100 (%) | 97.7 | 97.7 | 0.579 |
| Days of neutrophil engraftment, average (range) | 11.70 (9-19) | 11.91 (8-19) | 0.654 |
| Days of platelet engraftment, average (range) | 14.47 (9-31) | 16.09 (8-56) | 0.329 |

**Supplementary Table 3:** aGVHD, cGVHD and relapse after HSCT

|  | **HID + Unrelated (n=88)** | | |
| --- | --- | --- | --- |
|  | **ATG-T (10mg/kg, n=44)** | **ATG-F (20mg/kg, n=44)** | **P Value** |
| Cumulative incidence of aGVHD by day 100 (%) | 54.5 | 50.0 | 0.643 |
| Cumulative incidence of grade III-IV aGVHD by day 100 (%) | 15.9 | 15.9 | 0.983 |
| Overall grades of aGVHD, N (%) 1-2 3-4 | 17 (38.6) 7 (15.9) | 13 (29.5) 7 (15.9) | 0.681 |
| Cumulative incidence of cGVHD by 3 years (%) | 40.9 | 34.1 | 0.447 |
| Days of cGVHD onset, median (range) | 185.5(86-720) | 209 (104-875) | 0.362 |
| Severity of cGVHD, N (%)  no mild moderate severe | 26 (59.1) 3 (6.8) 10 (22.7) 5 (11.4) | 29 (65.9) 3 (6.8) 5 (11.4) 7 (15.9) | 0.532 |
| Days of relapse onset, median (range) | 205 (156-447) | 153 (86-253) | 0.085 |
| Cumulative incidence of relapse by 3 years (%) | 13.6 | 13.6 | 0.933 |

**Supplementary Table 4:** 3-year OS, 3-year CIR, TRM, 3-year DFS, 3-year GRFS after HSCT

|  | **HID + Unrelated (n=88)** | | |
| --- | --- | --- | --- |
|  | **ATG-T (10mg/kg, n=44)** | **ATG-F (20mg/kg, n=44)** | **P Value** |
| OS in 3 years, N (%) | 32 (72.7) | 31 (70.5) | 0.813 |
| Cumulative incidence of relapse by 3 years (%) | 13.6 | 13.6 | 0.933 |
| TRM within 100 days after transplantation, N (%) | 5 (11.4) | 4 (9.1) | 0.725 |
| Cumulative incidence of NRM by 3 years (%) | 20.5 | 25.0 | 0.720 |
| Overall incidence of DFS in 3 yeasr, N (%) | 29 (65.9) | 27 (61.4) | 0.658 |
| Overall incidence of GRFS in 3 years, N (%) | 22 (50.0) | 23 (52.3) | 0.831 |

OS, overall survival; TRM, non-relapse mortality within 100 days; NRM, non-relapse mortality; DFS, disease-free survival; GRFS, GVHD-free and relapse-free survival.

**Supplementary Table 5:** Univariate analysis

| **Univariate analysis** | **aGVHD ALL**  **HR (95% CI)**  **P Value** | **aGVHD Grade3-4**  **HR (95% CI)**  **P Value** | **cGVHD ALL**  **HR (95% CI)**  **P Value** | **Severe cGVHD**  **HR (95% CI)**  **P Value** |
| --- | --- | --- | --- | --- |
| Patient’s age:  <40 vs. ≥40 | 1.005 (0.115-8.758)  0.996 | 1.181 (0.254-5.498)  0.832 | 2.573 (0.879-7.531)  ***0.085*** | 3.754 (1.348-10.450)  ***0.011*** |
| Patient gender:  Female vs. Male | 6.541 (0.145-2.947)  0.580 | 1.856 (0.592- 5.82)  0.289 | 7.074 (0.256-1.953)  0.504 | 1.627 (0.616-4.297)  0.326 |
| Diagnosis:  AML vs. ALL vs. MDS vs. Others | 1.044 (0.332-3.276)  0.942 | 1.379 (0.617-3.083)  0.434 | 8.956 (0.480-1.669)  0.728 | 1.103 (0.646-1.882)  0.719 |
| Disease risk index:  Low/Intermediate risk vs.  High/Very high risk | 1.776 (0.353-8.922)  0.486 | 1.402 ( 0.477-4.116)  0.539 | 2.174 (0.615-7.685)  0.228 | 1.312 (0.496-3.470)  0.584 |
| Pre-transplant status:  NR vs. CR | 7.570 (0.000-Inf)  0.999 | 1.591 (0.446-5.666)  0.474 | 5.373 (0.183-1.576)  0.258 | 0.830 (0.299-2.303)  0.721 |
| HCT-CI: <2 vs. ≥2 | NA | NA | 2.629 (0.332-20.824)  0.360 | 4.048 (0.498-32.928)  0.191 |
| Stem cell source:  BM + PB vs. PB | 1.034 ( 0.243-4.399)  0.964 | 2.046 (0.752-5.570)  0.161 | 1.344 (0.500-3.607)  0.558 | 1.290 (0.533-3.123)  0.573 |
| Donor relatedness:  HID vs. Unrelated | 1.827 (0.395-8.451)  0.441 | 3.157 (1.033- 9.648)  ***0.044*** | 1.564 (0.574-4.266)  0.382 | 1.642 (0.668-4.037)  0.280 |
| Donor-recipient gender match:  Mismatch vs. Match | 9.006 (0.212-3.831)  0.887 | 0.713 (0.237-2.142)  0.547 | 7.419 (0.268-2.056)  0.566 | 1.021 (0.390-2.668)  0.967 |
| Donor-recipient ABO blood group match:  Mismatch vs. Match | 1.723 (0.415-7.158)  0.454 | 0.819 (0.298-2.247)  0.698 | 8.132 (0.295-2.242)  0.689 | 0.909 (0.359-2.301)  0.840 |
| Time from diagnose to transplantation:  <1 year vs. ≥1 year | 7.033 (0.082-6.024)  0.748 | 1.761 (0.482-6.432)  0.392 | 1.909 (0.600-6.067)  0.273 | 1.162 (0.379-3.556)  0.793 |
| The year of transplant:  <2018 vs. ≥2018 | 2.027 (0.404-10.164)  0.748 | 0.665 (0.238-1.859)  0.437 | 2.932 (0.929-9.251)  ***0.067*** | 0.968 (0.391-2.396)  0.945 |
| Use of letermovir:  Unused vs. Used | NA | NA | 1.087 (0.000-Inf)  0.998 | 0.000 (0.000-Inf)  0.998 |
| Conditioning regimen:  BU + CY vs. TBI + CY | 1.321 (0.000-Inf)  0.999 | 0.938 (0.205-4.279)  0.934 | 1.505 (0.195-11.615)  0.695 | 2.247 (0.279-18.102)  0.447 |
| ATG type:  ATG-F vs. ATG-T | 1.621 (0.376-6.990)  0.517 | 2.357 (0.842-6.597)  0.103 | 9.765 (0.358-2.662)  0.963 | 1.673 (0.682-4.105)  0.261 |

| **Univariate analysis** | **OS**  **HR (95% CI)**  **P Value** | **Relapse incidence**  **HR (95% CI)**  **P Value** | **NRM**  **HR (95% CI)**  **P Value** | **DFS**  **HR (95% CI)**  **P Value** | **GRFS**  **HR (95% CI)**  **P Value** |
| --- | --- | --- | --- | --- | --- |
| Patient’s age:  <40 vs. ≥40 | 1 (0.552-1.811)  1 | 2.751 (0.906-8.353)  ***0.074*** | 1.806 (0.370-8.803)  0.465 | 1.362 (0.730-2.543)  0.332 | 1.199 (0.619-2.324)  0.590 |
| Patient gender:  Female vs. Male | 1 (0.607-1.647)  1 | 2.131 (0.698-6.504)  0.184 | 0.376 (0.118-1.197)  ***0.098*** | 1.203 (0.703-2.059)  0.500 | 1.766 (0.963-3.239)  ***0.066*** |
| Diagnosis:  AML vs. ALL vs. MDS vs. Others | 1 (0.723-1.383)  1 | 1.768 (0.988-3.164)  ***0.055*** | 0.907 (0.346-2.376)  0.842 | 1.015 (0.725-1.422)  0.929 | 1.044 (0.704-1.549)  0.831 |
| Disease risk index:  Low/Intermediate risk vs.  High/Very high risk | 1 (0.607-1.647)  1 | 1.627 (0.577-4.591)  0.358 | 0.892 (0.280-2.841)  0.847 | 0.814 (0.479-1.384)  0.447 | 1.158 (0.643-2.085)  0.626 |
| Pre-transplant status:  NR vs. CR | 1 (0.509-1.965)  1 | 0.994 (0.323-3.057)  0.992 | 1.274 (0.277-5.860)  0.755 | 1.063 (0.521-2.170)  0.866 | 0.752 (0.350-1.617)  0.466 |
| HCT-CI: <2 vs. ≥2 | 1 (0.139-7.212)  1 | 3.881 (0.477-31.580)  0.205 | 0.000 (0.000-Inf)  0.999 | 1.280 (0.177-9.255)  0.807 | 0.000 (0.000-Inf)  0.998 |
| Stem cell source:  BM + PB vs. PB | 1 (0.609-1.641)  1 | 0.949 (0.354-2.543)  0.917 | 2.453 (0.775-7.760)  0.127 | 1.387 (0.814-2.365)  0.229 | 1.167 (0.644-2.115)  0.610 |
| Donor relatedness:  HID vs. Unrelated | 1 (0.592-1.688)  1 | 1.111 (0.385-3.207)  0.845 | 2.973 (0.894-9.886)  ***0.076*** | 1.147 (0.659-1.996)  0.627 | 1.128 (0.611-2.082)  0.700 |
| Donor-recipient gender match:  Mismatch vs. Match | 1 (0.609-1.641)  1 | 0.712 (0.256-1.978)  0.515 | 0.372 (0.107-1.291)  0.119 | 1.143 (0.674-1.937)  0.620 | 1.584 (0.877-2.862)  0.127 |
| Donor-recipient ABO blood group match:  Mismatch vs. Match | 1 (0.609-1.641)  1 | 1.443 (0.583-3.572)  0.427 | 0.534 (0.144-1.977)  0.348 | 1.074 (0.636-1.814)  0.791 | 1.234 (0.687-2.217)  0.482 |
| Time from diagnose to transplantation:  <1 year vs. ≥1 year | 1 (0.431-2.319)  1 | 1.236 (0.355-4.310)  0.739 | 1.207 (0.255-5.705)  0.813 | 0.823 (0.353-1.922)  0.653 | 1.227 (0.482-3.124)  0.668 |
| The year of transplant:  <2018 vs. ≥2018 | 1 (0.610-1.640)  1 | 0.868 (0.325-2.319)  0.777 | 0.612 (0.193-1.942)  0.405 | 0.618 (0.361-1.055)  ***0.078*** | 0.599 (0.332-1.081)  ***0.089*** |
| Use of letermovir:  Unused vs. Used | 1 (0.139-7.212)  1 | NA | NA | 1.280 (0.177-9.255)  0.807 | NA |
| Conditioning regimen:  BU + CY vs. TBI + CY | 1 (0.363-2.753)  1 | 1.328 (0.296-5.965)  0.711 | 0.000 (0.000-Inf)  0.998 | 1.297 (0.468-3.591)  0.617 | 1.463 (0.523-4.095)  0.469 |
| ATG type:  ATG-F vs. ATG-T | 1 (0.610-1.639)  1 | 1.595 (0.624-4.078)  0.330 | 1.582 (0.505-4.954)  0.431 | 1.320 (0.779-2.237)  0.302 | 1.004 (0.560-1.802)  0.989 |

| **Univariate analysis** | **Cytomegalovirus reactivation**  **HR (95% CI)**  **P Value** | **Epstein–Barr virus reactivation**  **HR (95% CI)**  **P Value** | **Bacterial infection**  **HR (95% CI)**  **P Value** | **Fungus infection**  **HR (95% CI)**  **P Value** | **Other virus**  **infection**  **HR (95% CI)**  **P Value** |
| --- | --- | --- | --- | --- | --- |
| Patient’s age:  <40 vs. ≥40 | 4.827 (0.793-29.390 )  ***0.088*** | 2.932 (0.970-8.860)  ***0.057*** | 2.630 (0.363-19.067)  0.339 | 2.588 (0.641-10.456)  0.182 | 6.486 (1.833-22.946)  ***0.004*** |
| Patient gender:  Female vs. Male | 1.201 (0.218-6.599)  0.833 | 1.372 (0.536-3.511)  0.510 | 1.689 (0.177-16.127)  0.649 | 1.708 (0.530-5.503)  0.369 | 2.113 (0.679-6.577)  0.197 |
| Diagnosis:  AML vs. ALL vs. MDS vs. Others | 1.367 (0.433-4.315)  0.594 | 1.368 (0.786-2.380)  0.268 | 7.590 (0.218-2.648)  0.666 | 1.757 (0.807-3.826)  0.156 | 2.066 (1.115-3.827)  ***0.021*** |
| Disease risk index:  Low/Intermediate risk vs.  High/Very high risk | 1.706 (0.327-8.905)  0.526 | 1.241 (0.507-3.036)  0.636 | 9.330 (0.095-9.204)  0.953 | 1.356 (0.277-6.649)  0.707 | 1.073 (0.393-2.929)  0.891 |
| Pre-transplant status:  NR vs. CR | 2.956 (0.000-Inf)  0.999 | 1.115 (0.409-3.044)  0.831 | 2.240 (0.014-3.590)  0.290 | 1.858 (0.403-8.571)  0.427 | 1.216 (0.339-4.364)  0.764 |
| HCT-CI: <2 vs. ≥2 | 4.162(0.383-45.229)  0.241 | 2.790 (0.343-22.728)  0.338 | 1.072 (0.109-10.574)  0.953 | 0.000 (0.000-Inf)  0.999 | 4.957 (0.571-43.054)  0.147 |
| Stem cell source:  BM + PB vs. PB | 1.457(0.320-6.631)  0.626 | 1.617 (0.701-3.730)  0.260 | 1.215 (0.200-7.394)  0.832 | 1.739 (0.517-5.843)  0.371 | 1.738 (0.647-4.672)  0.273 |
| Donor relatedness:  HID vs. Unrelated | 9.240 (1.014-84.244)  ***0.049*** | 2.408 (0.933-6.217)  ***0.069*** | 7.100 (0.113-4.452)  0.714 | 1.787 (0.514-6.212)  0.361 | 2.866 (0.864-9.505)  ***0.085*** |
| Donor-recipient gender match:  Mismatch vs. Match | 1.106 (0.235-5.202)  0.899 | .0.592 (0.232-1.514)  0.274 | 9.910 (0.000-Inf)  0.999 | 1.154 (0.341-3.903)  0.818 | 0.804 (0.275-2.356)  0.691 |
| Donor-recipient ABO blood group match:  Mismatch vs. Match | 1.352(0.294-6.220)  0.698 | 0.943 (0.414-2.152)  0.890 | 8.230 (0.135-5.006)  0.832 | 0.855 (0.294-2.485)  0.774 | 1.115 (0.428-2.906)  0.823 |
| Time from diagnose to transplantation:  <1 year vs. ≥1 year | 4.855 (0.436-54.004)  0.199 | 1.283 (0.465-3.541)  0.631 | 5.920 (0.062-5.654)  0.649 | 0.499 (0.108-2.292)  0.371 | 1.836 (0.510-6.602)  0.352 |
| The year of transplant:  <2018 vs. ≥2018 | 6.610 (0.146-2.998)  0.591 | 0.677 (0.272-1.683)  0.401 | 9.330 (0.095-9.204)  0.953 | 0.699 (0.171-2.848)  0.617 | 0.613 (0.215-1.746)  0.359 |
| Use of letermovir:  Unused vs. Used | NA | NA | NA | NA | NA |
| Conditioning regimen:  BU + CY vs. TBI + CY | 0.000 (0.000-Inf)  0.999 | 0.916 (0.209-4.003)  0.907 | 4.472 (0.279-71.807)  0.290 | 7.278 (0.655-80.917)  0.106 | 1.451 (0.318-6.633)  0.631 |
| ATG type:  ATG-F vs. ATG-T | 7.990 (0.174-3.658)  0.772 | 2.054 (0.864-4.886)  0.103 | 3.348 (0.336-33.362)  0.303 | 8.073 (1.902-34.266)  ***0.005*** | 1.635 (0.568-4.708)  0.363 |

**Supplementary Table 6:** Multivariate Analyses

| **Multivariate analysis** | **cGVHD ALL**  **HR (95% CI)**  **P Value** |
| --- | --- |
| Patient’s age:  <40 vs. ≥40 | 0.401 (0.141-1.14)  0.087 |
| The year of transplant:  <2018 vs. ≥2018 | 0.651 (0.336-1.26)  0.200 |

| **Multivariate analysis** | **Relapse incidence**  **HR (95% CI)**  **P Value** |
| --- | --- |
| Patient’s age:  <40 vs. ≥40 | 0.716 (0.146-3.51)  0.68 |
| Diagnosis:  AML vs. ALL vs. MDS vs. Others | 0.617 (0.239-1.59)  0.32 |

| **Multivariate analysis** | **NRM**  **HR (95% CI)**  **P Value** |
| --- | --- |
| Patient gender:  Female vs. Male | 1.962 (0.689-5.59)  0.21 |
| Donor relatedness:  HID vs. Unrelated | 0.735 (0.248-2.18)  0.58 |

| **Multivariate analysis** | **GRFS**  **HR (95% CI)**  **P Value** |
| --- | --- |
| Patient gender:  Female vs. Male | 1.488 (1.048-2.11)  **0.026** |
| The year of transplant  <2018 vs. ≥2018 | 0.801 (0.548-1.17)  0.250 |

| **Multivariate analysis** | **Cytomegalovirus reactivation**  **HR (95% CI)**  **P Value** |
| --- | --- |
| Patient’s age:  <40 vs. ≥40 | 0.545 (0.272-1.09 )  0.087 |
| Donor relatedness:  HID vs. Unrelated | 0.751 (0.416-1.36)  0.340 |

| **Multivariate analysis** | **Epstein–Barr virus reactivation**  **HR (95% CI)**  **P Value** |
| --- | --- |
| Patient’s age:  <40 vs. ≥40 | 3.75 (0.328-42.9)  0.29 |
| Donor relatedness:  HID vs. Unrelated | 2.29 (0.201-26.3)  0.50 |

| **Multivariate analysis** | **Other virus**  **infection**  **HR (95% CI)**  **P Value** |
| --- | --- |
| Patient’s age:  <40 vs. ≥40 | 0.252 (0.0592-1.07)  0.062 |
| Diagnosis:  AML vs. ALL vs. MDS vs. Others | 0.950 (0.5435-1.66)  0.860 |
| Donor relatedness:  HID vs. Unrelated | 0.625 (0.2720-1.43)  0.270 |
